# Exogenous cytokinin application to *Actinidia chinensis* var. *deliciosa* ‘Hayward’ fruit promotes fruit expansion through water uptake

**DOI:** 10.1038/hortres.2017.43

**Published:** 2017-09-20

**Authors:** Simona Nardozza, Helen L Boldingh, Mark W Wohlers, Andrew P Gleave, Zhiwei Luo, Guglielmo Costa, Elspeth A MacRae, Michael J Clearwater, Annette C Richardson

**Affiliations:** 1 The New Zealand Institute for Plant & Food Research Limited (PFR), Mt Albert Research Centre, Private Bag 92169, Auckland, New Zealand; 2 PFR, Ruakura Research Centre, Private Bag 3123, Hamilton, New Zealand; 3 Dipartimento di Scienzie Agrarie, Università di Bologna, Via Fanin 46, Bologna 40127, Italy; 4 PFR, Te Puke Research Centre, Te Puke 3182, New Zealand; 5 PFR, Kerikeri Research Centre, Private Bag 23, Kerikeri, New Zealand

## Abstract

Exogenous application of a cytokinin-like compound forchlorfenuron (CPPU) can promote fruit growth, although often at the expense of dry matter (DM), an important indicator of fruit quality. *Actinidia chinensis* var. *deliciosa* ‘Hayward’ fruit are very responsive to CPPU treatments, but the mechanism underlying the significant fruit weight increase and associated decrease in DM is unclear. In this study, we hypothesised that CPPU-enhanced growth increases fruit carbohydrate demand, but limited carbohydrate supply resulted in decreased fruit DM. During fruit development, CPPU effects on physical parameters, metabolites, osmotic pressure and transcriptional changes were assessed under conditions of both standard and a high carbohydrate supply. We showed that CPPU increased fruit fresh weight but the dramatic DM decrease was not carbohydrate limited. Enhanced glucose and fructose concentrations contributed to an increase in soluble carbohydrate osmotic pressure, which was correlated with increased water accumulation in CPPU-treated fruit and up-regulation of water channel aquaporin gene *PIP2.4* at 49 days after anthesis. Transcipt analysis suggested that the molecular mechanism contributing to increased glucose and fructose concentrations was altered by carbohydrate supply. At standard carbohydrate supply, the early glucose increase in CPPU fruit was associated with reduced starch synthesis and increased starch degradation. When carbohydrate supply was high, the early glucose increase in CPPU fruit was associated with a general decrease in starch synthesis but up-regulation of vacuolar invertase and fructokinase genes. We conclude that CPPU affected fruit expansion by increasing the osmotically-driven water uptake and its effect was not carbohydrate supply-limited.

## Introduction

Fruit size (fresh weight) and dry matter (DM, defined as g of dry weight per kg of fresh weight) are important quality traits for horticultural crops affecting their value, determining their economical returns and driving consumer purchases.^1^ Fruit fresh weight increase can be achieved by thinning, although this intervention can decrease the potential yield.^2^ Plant growth regulators are another tool available to increase fruit fresh weight and boost yield, which often appeals to growers. However, a number of studies have shown how the use of growth regulators can negatively affect fruit quality.^3–5^ The growth regulator forchlorfenuron (*N*_1_-(2-chloro-4-pyridyl)-*N*_3_-phenylurea, CPPU) is an example of a synthetic cytokinin that influences fruit set and/or development of several fruiting crop species, such as apples,^6^ grapes,^7^ watermelon,^8^ *Lagenaria leucantha*,^9^ macadamia^10^ and kiwi fruit.^11,12^

While a large number of studies have been conducted in kiwifruit^5,11–19^ using CPPU, there has been no clear explanation of its molecular mechanism in any species. These studies agree that CPPU application to kiwifruit dramatically increased fruit fresh weight but this desired effect was often accompanied by an undesirable decrease in final fruit DM.^5^ Antognozzi *et al.*^11^ also found CPPU-treated fruit to be lower in DM, despite having higher carbohydrate concentration (starch, glucose and fructose) and enhanced starch metabolism. Patterson *et al.*^5^ showed CPPU promoted fruit fresh weight through cell expansion and Ainalidou *et al.*^20^ confirmed it affected small cell size, rather than by increasing cell division as later observed by Cruz-Castillo *et al.*,^16^ despite having a similar timing of application. Together, these findings suggest that CPPU has a role in the modulation of both carbohydrate metabolism and water accumulation.

Key carbohydrate metabolic genes, such as ADP-glucose pyrophosphorylase large subunit 4 (*APL4*) and beta-amylase 9 (*BAM9*), important during kiwifruit development and DM accumulation, were previously identified and their transcripts investigated in genotypes with contrasting carbohydrate metabolism.^21^ In high starch and high DM genotypes, the higher and extended transcription of *APL4* was associated with enhanced starch concentration in fruit, whilst an involvement of *BAM9* in starch turnover was proposed during early fruit development.

The role of aquaporins, transmembrane water channel proteins that facilitate water transport across membranes,^22^ has been described for several fruit species.^23–25^ Of the seven plant aquaporin subfamilies,^26^ only plasma membrane intrinsic proteins (PIP) have been attributed to having a large role in controlling water movement across membranes.^27^ These proteins may also contribute to cell osmotic adjustments following continuous sugar accumulation and cell expansion during fruit growth.^28,29^ The molecular mechanisms of water accumulation have not been investigated in kiwifruit to date.

Despite the low DM observed in CPPU-treated fruit, current literature suggests that CPPU increases fruit sink strength (the capacity to attract carbohydrates^30^) promoting carbohydrate accumulation in the fruit but that the dramatic fruit fresh weight increase is not well supported by carbohydrate supply from leaves.^5,19^ In conditions of high carbohydrate supply, kiwifruit vines were able to redistribute carbohydrates to fruit, including to fruit growing on leafless shoots.^31^ Experiments have shown that on average between two to three leaves were required to satisfy the normal carbohydrate demand of a fruit,^32^ and on a whole plant basis fruit fresh weight increased up to a leaf-to-fruit ratio of five.^33^

The aim of this study was to find what limits kiwifruit DM accumulation after CPPU application. We proposed that carbohydrate supply limitation could be the cause of low DM for CPPU-treated fruit. The leaf-to-fruit ratio of girdled 1-year-old fruiting canes was adjusted to test the effect of two different controlled carbohydrate supplies (standard and high) on CPPU treatment. We verified that CPPU-treated fruit had higher fruit weights and lower DM. We hypothesised that the lower DM was the result of a reduction in starch concentration. We then investigated how the changes in the fresh weight growth and carbohydrate metabolism in the CPPU-treated fruit were modulated by examining transcriptional variation of genes involved in metabolism and/or movement of carbohydrates and water transport.

## Materials and methods

### Plant material

The experiment was carried out on *Actinidia chinensis* var. *deliciosa* (A. Chev.) A. Chev. ‘Hayward’ 15-year-old vines planted in an orchard block at Plant & Food Research (PFR), Te Puke Research Centre (Bay of Plenty, NZ; 37° 49′ S 176° 19’ E). All vines were grown on clonal *Actinidia hemsleyana* Dunn ‘Kaimai’ rootstocks. The vines were managed for normal commercial production (except for pruning, thinning and girdling of the experimental canes) on a pergola-trained system with 6 m spacing within the row and 5 m spacing between rows.^34^ The date of anthesis (defined as 50% open flowers) was recorded as the 23rd of November. Treatment and sampling dates were referred to as days after anthesis (DAA).

### Treatments

The experiment was a two by two factorial design with equal numbers of canes allocated to two carbohydrate supplies (standard and high) by two CPPU treatments (treated and untreated). A total of 120 uniform 1-year-old canes (8 canes per vine) were selected. Five canes per treatment were randomly allocated to five sampling times. All canes were girdled at 28 DAA and all the leaves and fruit distal from the girdle counted. CPPU was applied to fruitlets 28 DAA as a 10 ppm dip. On the same day the leaf-to-fruit ratio was adjusted on girdled canes to either three leaves per fruit (standard carbohydrate supply) or six leaves per fruit (high carbohydrate supply). The girdles were kept open throughout the experiment and leaf-to-fruit ratios maintained by immediately removing any new growth on canes.

### Sampling

Fruit samples were collected 35, 49, 70, 126 and 154 DAA. At each sampling date six randomly selected fruit were harvested from each of five selected canes per treatment. Fruit fresh weight, fruit dry weight and DM were measured on all fruit as described in Nardozza *et al.*^35^ DM, the ratio of fruit dry weight to fruit fresh weight, was determined by drying 2-mm fruit slices at 65 °C for 24 h^36^ and expressed as g kg^−1^. Mean absolute growth rate (AGR) was calculated for fruit fresh and dry weights as described by Opara.^37^

Fruit samples, consisting of longitudinal slices representative of all fruit tissue types, were collected from four biological replicates, snap-frozen in liquid nitrogen, and stored at −80 °C for carbohydrate and transcript analysis.

### Non-structural carbohydrate and organic acid analysis

Starch was colourimetrically determined following enzymatic digestion as reported by Smith *et al.*^38^ Soluble carbohydrates (glucose, fructose, sucrose, *myo*-inositol and galactose) were analysed as per Klages *et al.*^39^ and organic acids as per Cheng *et al.*^40^ by gas chromatography (Carlo Erba GC 6000) with a DB1701 column and FID detection. The individual starch, sugar and acid contents were expressed as milligrams per gram fresh fruit weight (mg gFW^−1^). Osmotic pressures were calculated as described in Nardozza *et al.*^35^ Briefly, the contribution of soluble carbohydrates and organic acids to total fruit osmotic pressure were calculated from the solute concentrations applying the van‘t Hoff relation^41^ with the same conditions described by Bertin *et al.*^42^

### Transcript analysis

Following RNA isolation from 2 g of fruit tissues,^43^ DNase treatment (DNA-free™, Ambion-Invitrogen) and cDNA synthesis (SuperScript™ III Reverse Transcriptase, Invitrogen), transcript analysis was performed by real-time PCR on a LightCycler 480 detection system (Roche) using LightCycler 480 SYBR Green master mix (Roche) as described by Nardozza *et al.*^21^ Gene-specific primers, designed to span exon junctions, were used as reported by Nardozza *et al.*^21^ or as listed in Supplementary Table S1. Target gene transcripts were normalised to the housekeeping genes elongation factor 1α, *EF1α*, and protein phosphatase 2A, *PPRSA*.

### Statistical analysis

The effect of CPPU treatment, carbohydrate supply and fruit age on fruit fresh weight, fruit dry weight, DM, starch, glucose, fructose and sucrose concentrations, total starch content per fruit, osmotic pressure and DM components were analysed by a general three-way factorial ANOVA using GenStat software version 17.1.0.14731 (VSN International Ltd, UK). For variables characterised by large developmental changes, such as fruit fresh weight, fruit dry weight, dry matter and starch, the data analysis considering fruit age as a factor produced standard errors that were larger than the measured value at 35 DAA (7 days after the treatment), therefore hiding significant 2-fold variations between treatments. For this reason, the effect of CPPU treatment and carbohydrate supply on fruit fresh weight, fruit dry weight, DM and starch concentration at 35 DAA were also tested using a two-way ANOVA.

## Results

### CPPU increased both fresh and dry fruit weight

**Figure 1:**
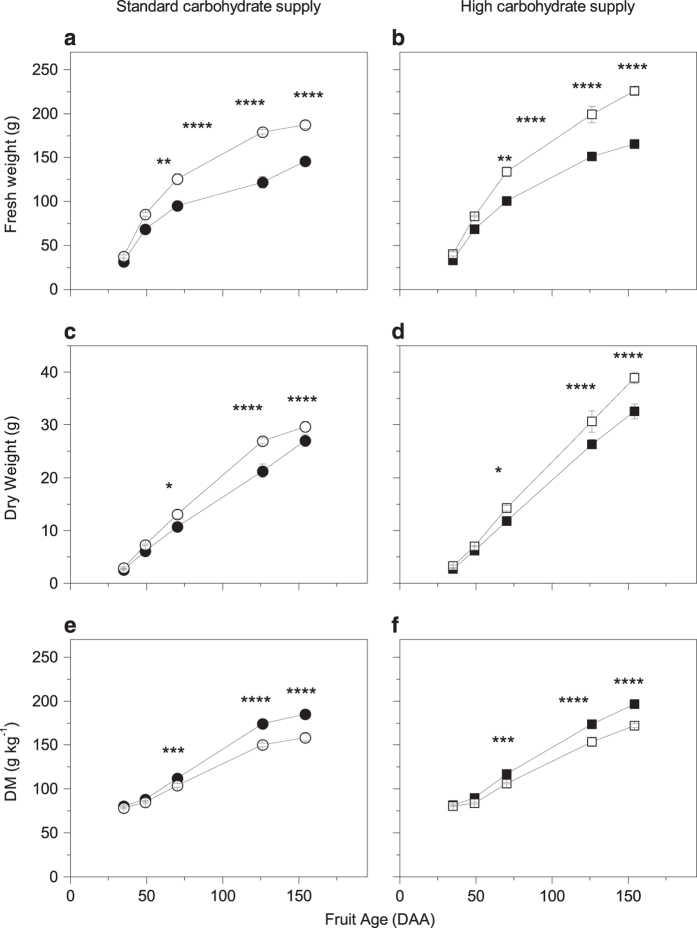
Effects of CPPU on growth in fruit fresh weight (**a**, **b**), dry weight (**c**, **d**) and dry matter (DM; **e**, **f**) of ‘Hayward’ kiwifruit grown with two different carbohydrate supplies. Circles represent standard carbohydrate supply and squares represent high carbohydrate supply. Closed symbols are untreated fruit and opened symbols are CPPU-treated fruit. Values are averages ±s.e. of the mean. *n*=5. Three-way factorial ANOVA: *P<0.05; **P<0.01; ***P<0.001; ****P<0.0001; blank, not significant. DAA, days after anthesis.

CPPU significantly affected kiwifruit fruit weight, at both standard and high carbohydrate supply. The CPPU effect on fruit fresh weight was evident from 49 DAA (~20 days after the treatment) through to harvest for fruit from both carbohydrate supplies. CPPU-treated fruit had a final fruit fresh weight 28 percent greater than untreated fruit with a standard carbohydrate supply, and 37 percent greater than untreated fruit with a high carbohydrate supply (Figures 1a and b).

Fruit dry weight was also affected by CPPU treatment, being higher in CPPU-treated fruit, with both carbohydrate supplies. Dry weight differences were detectable from 70 DAA (~40 days after the treatment) until final harvest. The relative change in dry weight accumulation with CPPU treatment was less than the effect on fresh weight (10 percent and 20 percent by harvest at standard and high carbohydrate supply respectively; Figures 1c and d).

By final harvest, CPPU treatment led to an increase in 38.6 g H_2_O/fruit (38 percent) and 54.3 g H_2_O/fruit (41 percent) at the standard and high carbohydrate supply, respectively.

### CPPU reduced fruit DM

Differences in DM were first detectable at 70 DAA (~40 days after the treatment) and continued through until harvest. At the final harvest, fruit DM was significantly lowered by approximately 25 g kg^−1^ by CPPU treatment with both carbohydrate supplies (185±4 g kg^−1^ vs. 159±4 g kg^−1^ standard supply and 197±3 g kg^−1^ vs. 172±2 g kg^−1^ high supply for untreated and CPPU-treated fruit respectively; *P*<0.0001; Figures 1e and f). Conversely, at the final harvest, high carbohydrate supply had a positive effect in enhancing fruit DM by approximately 12 g kg^−1^ compared to fruit from the low carbohydrate supply (*P*<0.0001).

### CPPU increased fruit glucose and fructose concentrations but reduced starch concentration

**Figure 2:**
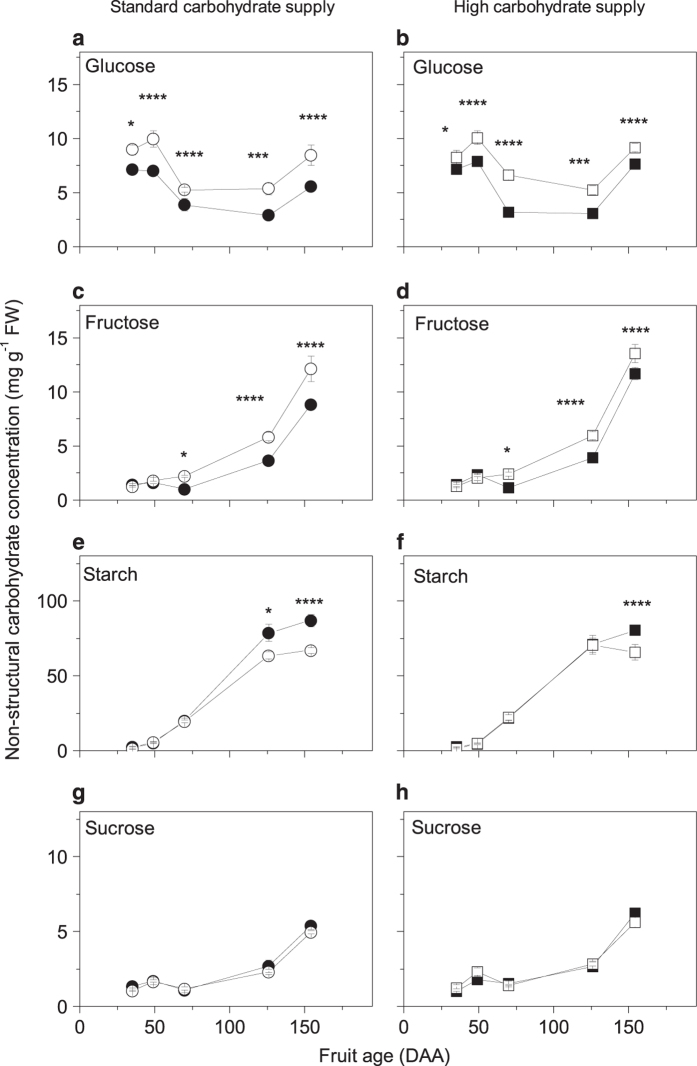
Effects of CPPU on concentrations of glucose (**a**, **b**), fructose (**c**, **d**), starch (**e**, **f**) and sucrose (**g**, **h**) on ‘Hayward’ kiwifruit grown with two different carbohydrate supplies. Circles represent standard carbohydrate supply and squares represent high carbohydrate supply. Closed symbols are untreated fruit and opened symbols are CPPU-treated fruit. Values are averages ±s.e. of the mean. *n*=4. Three-way factorial ANOVA: *P<0.05; **P<0.01; ***P<0.001; ****P<0.0001; blank, not significant. DAA, days after anthesis.

CPPU significantly increased glucose concentration in fruit by 1.3–1.8-fold and the effect was evident at 35 DAA (7 days after the treatment) until final harvest, irrespective of carbohydrate supply (Figures 2a and b). Treated fruit also showed a significant twofold increase in fructose concentration but the effects were only evident at 70 DAA until harvest (Figures 2c and d). The three-way ANOVA analysis showed CPPU only had a consistent and significant effect of decreasing fruit starch concentration at the final harvest (154 DAA; Figures 2e and f). However, on a per fruit basis, total starch content was significantly increased by CPPU treatment at 126 DAA, particularly at high carbohydrate supply (10.7±1.1 g starch/fruit and 14.0±1.0 g starch/fruit in untreated and CPPU-treated fruit respectively; *P*=0.03, fruit age x treatment interaction). Sucrose (Figures 2g and h), galactose, *myo*-inositol, malic, citric and quinic acids concentrations (Supplementary Table S2) were unaffected by CPPU treatment or carbohydrate supply.

A two-way ANOVA analysis carried out on small young fruit at 35 DAA (7 days after the treatment) also showed significant treatment affects. CPPU-treated fruit were significantly larger, had higher dry weights and lower starch concentrations than untreated fruit (Table 1), whilst DM was significantly decreased by CPPU treatment and increased by greater carbohydrate supply. This decrease in DM in CPPU-treated fruit was due to a halving of starch concentrations compared with those in untreated fruit.

### CPPU effects on DM components

Soluble carbohydrates represented a consistently and significantly higher proportion of DM in CPPU-treated fruit (*P*<0.001; Table 2, Supplementary Table S3 and Supplementary Figure S1) throughout fruit development. CPPU treatment significantly reduced the organic acid component of DM (*P*=0.026) whilst it had no overall and consistent effect on starch and other components of DM. However, it is worth noting that between 126 and 154 DAA the starch component decreased in CPPU-treated fruit with high carbohydrate supply (Table 2 and Supplementary Figure S1d), whilst it was steady in all the other treatments (Supplementary Figures S1a–c). This suggests that an early onset of fruit maturation occurred in CPPU-treated fruit with high carbohydrate supply.

### CPPU treatment altered the soluble carbohydrates component of the fruit osmotic pressure

**Figure 3:**
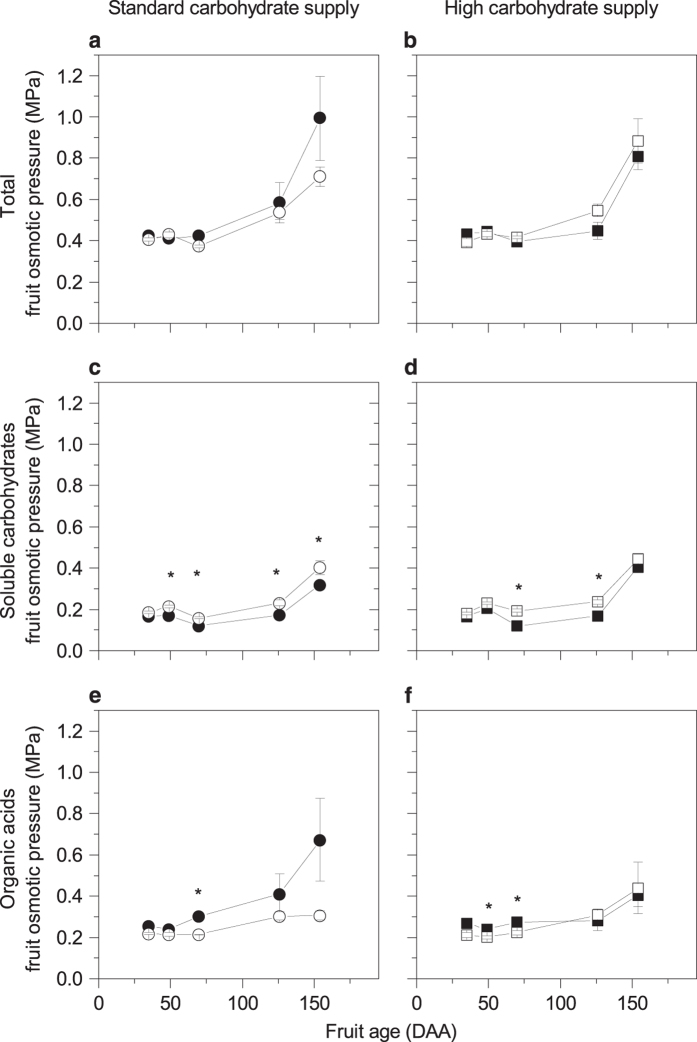
Effects of CPPU on total fruit osmotic pressure (**a**, **b**), soluble carbohydrates osmotic pressure (**c**, **d**) and organic acids osmotic pressure (**e**, **f**) on ‘Hayward’ kiwifruit grown with two different carbohydrate supplies. Circles represent standard carbohydrate supply and squares represent high carbohydrate supply. Closed symbols are untreated fruit and opened symbols are CPPU-treated fruit. Values are averages ±s.e. of the mean. *n*=4. Three-way factorial ANOVA: *P<0.05; blank, not significant. DAA, days after anthesis.

CPPU had a significant effect on the soluble carbohydrates component of the fruit osmotic pressure (Figures 3c and d; Table 3). There was also a significant interaction between fruit age and carbohydrate supply. In fruit with standard carbohydrate supply the soluble carbohydrates component of the fruit osmotic pressure was significantly greater from 49 DAA onwards, whereas in fruit with high supply it was only significantly higher at 70 and 126 DAA (~40 and 100 days after the treatment).

### CPPU effects on genes involved in carbohydrate metabolism, and the movement of soluble carbohydrate and water

**Figure 4:**
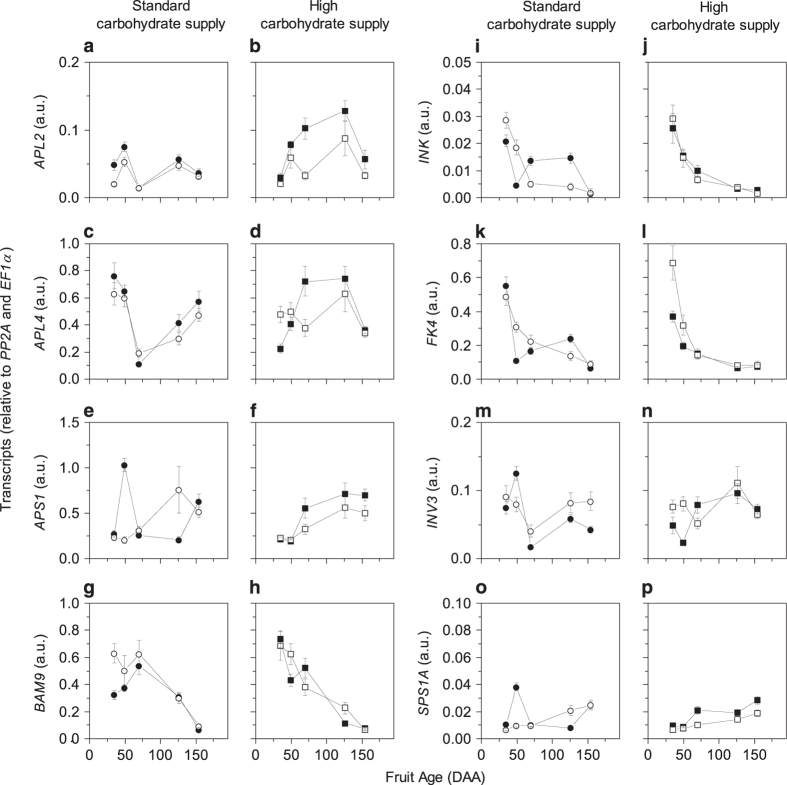
Transcript abundance of kiwifruit carbohydrate metabolism genes showing variation associated with CPPU treatment at a standard and a high carbohydrate supply during development of ‘Hayward’ fruit: ADP-glucose pyrophosphorylase large subunit 2 (*APL2*; **a**, **b**), ADP-glucose pyrophosphorylase large subunit 4 (*APL4*; **c**, **d**), ADP-glucose pyrophosphorylase small subunit 1 (*APS1*; **e**, **f**), beta-amylase 9 (*BAM9*; **g**, **h**), neutral invertase K (*INK*; **i**, **j**), fructokinase 4 (*FK4*; **k**, **l**), vacuolar invertase 3 (*INV3*; **m**, **n**), sucrose phosphate synthase 1A (*SPS1A*; **o**, **p**). Circles represent standard carbohydrate supply and squares represent high carbohydrate supply. Closed symbols are untreated fruit and opened symbols are CPPU-treated fruit. Values are averages ±SE of the mean. *n*=4. a.u., arbitrary unit; DAA, days after anthesis.

Transcript analysis for genes involved in carbohydrate (starch and soluble carbohydrates) metabolism, as well as sugar and water movement (sugar transporters and aquaporins, respectively), showed that application of CPPU and/or high carbohydrate supply contributed to a variation in transcript abundance of some genes in comparison to the untreated control with a standard carbohydrate supply (Supplementary Figure S2; Figure 4). In contrast with what has been shown with growth and metabolite data described in Figures 1 and 2, we did not observe a common pattern for transcripts in CPPU-treated fruit with different carbohydrate supplies. For this reason, we described the results for carbohydrate metabolism and movement separately starting with the standard carbohydrate supply for ‘Hayward’ kiwifruit. CPPU-treated fruit with a standard carbohydrate supply had twofold decrease in *APL2* (ADP-glucose pyrophosphorylase large subunit 2, involved in starch synthesis; Figure 4a) transcription and a twofold increase in *BAM9* (beta-amylase 9, possibly involved in starch degradation; Figure 4g) transcription at 35 DAA (7 days after the treatment). At 49 DAA, CPPU-treated fruit had a 4-fold and 3-fold increase in *INVK* (neutral invertase K; Figure 4j) and *FK4* (fructokinase 4; Figure 4l) transcript levels, respectively. By 70 DAA, transcription of *INV3* (vacuolar acid invertase 3; Figure 4n) increased twofold (and these higher transcripts were maintained until harvest).

When fruit had a high carbohydrate supply, there was a general decrease in transcription of all ADP-glucose pyrophosphorylase genes tested (*APL2*, *APL4*, large subunit 4 and *APS1*, small subunit 1; Figures 4b, d and f) throughout fruit development in CPPU-treated fruit. Early in fruit development, transcript levels for *FK4* (Figure 4m) and *INV3* (Figure 4o) were higher in CPPU-treated samples, and by 49 DAA *INV3* transcription was fourfold higher than the control. From 70 DAA onwards, sucrose phosphate synthase A1 (*SPSA1;* Figure 4q) transcripts were 1.5–2-fold lower in CPPU-treated fruit than in untreated fruit, and sucrose synthase 1 (*SUS1*; Supplementary Figure S2) was also down-regulated in CPPU-treated fruit. In CPPU-treated fruit with a high carbohydrate supply, transcription of *BAM3* (a beta-amylase isoform mainly expressed at harvest and during ripening) started at 126 DAA, earlier than in fruit from other treatments (data not shown due to low transcription at early stages), suggesting an early onset of maturation in these fruit. This is further supported by the significantly higher total soluble carbohydrate concentration of CPPU-treated fruit from 126 DAA onwards (*P*<0.001; Table 2 and Supplementary Table S3).

**Figure 5:**
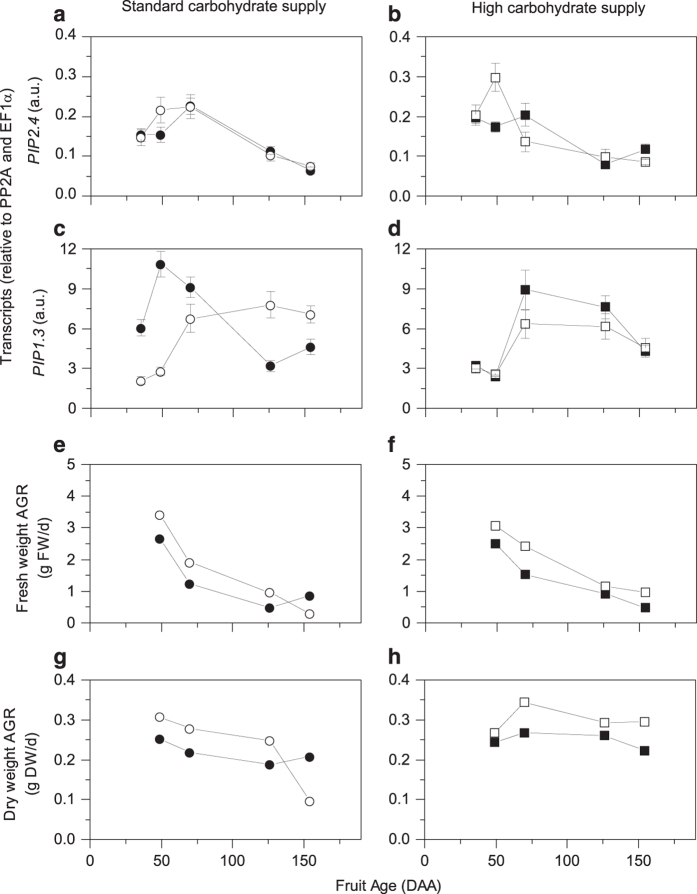
Aquaporin *PIP2.4* (**a**, **b**) and *PIP1.3* (**c**, **d**) transcripts, and absolute growth rates (AGR) for fresh weight (**e**, **f**) and dry weight (**g**, **h**) during development of ‘Hayward’ fruit grown at two different carbohydrate supplies and with or without CPPU treatment. Circles represent standard carbohydrate supply and squares represent high carbohydrate supply. Closed symbols are untreated fruit and opened symbols are CPPU-treated fruit. Values are averages ±s.e. of the mean. *n*=4. a.u., arbitrary unit; DAA, days after anthesis.

At 49 DAA the water channel protein *PIP2.4* transcription was 1.4- and 1.7-fold higher in CPPU-treated fruit (at standard and high carbohydrate supply, respectively) (Supplementary Figure S2 and Figures 5a and b). Transcript levels for *PIP1.3* peaked at 49 DAA and then decreased throughout the rest of fruit development (Supplementary Figure S2 and Figures 5c and d). With the application of CPPU and/or high carbohydrate supply, the peak in *PIP1.3* transcription was delayed to 70 DAA and the transcription decreased more slowly than the untreated fruit at standard supply (Figures 5c and d). These transcript accumulation patterns were not reflected by changes in fresh weight AGR, but mirrored the dry weight AGR (Figures 5e–h).

## Discussion

Exogenous cytokinin treatment affected ‘Hayward’ fruit development as expected by increasing fresh weight. An unexpected result was the inability of doubling of carbohydrate supply to compensate for the 25 g kg^−1^ reduction in DM caused by CPPU application to fruit. The reduction in DM was the result of a higher water accumulation in CPPU-treated fruit osmotically driven by increased glucose and fructose concentrations, through a molecular mechanism influenced by carbohydrate supply.

### CPPU increased fruit fresh weight

The most obvious and widely reported CPPU effect is the dramatic increase in fruit fresh weight, for kiwifruit as well for other fruit crops.^5,7,8,11–13,15,18,19,44–46^ The observed 28 and 37% increases in fresh weight at standard and high carbohydrate supply, respectively, are in agreement with previous observations. However, the controlled system created by application of girdles enhanced the average fruit weight from all treatments, with the untreated fruit at standard carbohydrate supply having a fresh weight of about 150 g, which is higher than commercially available kiwifruit^47^ The combination of both CPPU and high carbohydrate supply resulted in a 54 percent higher fresh weight, which equated to an average fruit weight of 225 g, a figure that is nearly double that of a commercially available kiwifruit^47^ and probably close to the maximum potential size for these fruit.

### Increasing carbohydrate supply did not prevent DM loss in CPPU-treated fruit

Isolating the cane by girdling (and thus preventing photosynthate redistribution from other parts of the vine) also amplified the effect of CPPU on lowering fruit DM at both carbohydrate supplies, with a much greater decrease than reported in previous studies.^5,11^ The higher carbohydrate supply was not sufficient to supply the fruit with adequate photosynthate to balance the dramatic fruit fresh weight increase promoted by CPPU treatment, and DM was decreased by 25 g kg^−1^ DM with either carbohydrate supply. This undesirable reduced DM ‘side-effect’ of CPPU treatment was consistent with previous studies.^5,11^ In a commercial orchard in New Zealand, a 25 g kg^−1^ DM decrease by a CPPU treatment would translate in to large financial loss per hectare (Bill Snelgar, personal communication) through reduced DM quality payments.^48^

Using whole vine manipulations, Richardson *et al.*^49^ showed that a 50% increase in carbohydrate supply led to an enhancement in both fruit fresh weight and DM, although DM was increased by only 4–5 g kg^−1^. However, in our study, where girdling prevented carbohydrate redistribution from canes to the rest of the vine,^31^ doubling carbohydrate supply increased fruit DM by 12 g kg^−1^ in both CPPU-treated and untreated fruit.

### DM reduction was not only the result of lower starch concentration

The findings of our experiment showed a reduction of starch concentration and DM at 35 DAA (7 days after CPPU application) in CPPU-treated fruit. In CPPU-treated fruit with a standard carbohydrate supply, apart from 35 DAA starch concentration was unaffected until later in development (126 DAA onwards), when the lower DM reflected the decrease in starch concentration. In fruit from high carbohydrate supply, starch concentration did not differ between treated and untreated fruit from 35 DAA until harvest, when the lower starch in CPPU-treated fruit was probably due to an early onset of fruit maturation, as previously observed by Patterson *et al.*^5^ Starch is one of the major components of DM in *Actinidia chinensis* var. *deliciosa* fruit, representing about 40% of the total DM at harvest time.^35^ Our data (Table 1, Figures 1 and 2) clearly showed a negative relationship between CPPU application and DM, and starch concentration was lowered by CPPU treatment at certain fruit developmental stages. Our results are in disagreement with one previously published study where CPPU increased starch concentration in kiwifruit,^11^ although the starch data was not statistically analysed and contradicts a reduction in fruit DM in that study. As starch is a major component of DM,^35^ we would expect starch to decrease with decreasing DM as it did in our study.

### CPPU-led enhancement of glucose and fructose

Combining the transcript accumulation data and the carbohydrate analysis, we propose two models for carbohydrate metabolism following CPPU treatment under standard or high carbohydrate supply. In our study, carbohydrate supply treatments contributed to the variation of transcript accumulation, as previously observed in citrus.^50^ For this reason, we will discuss the two models separately.

At 35 DAA (7 days after the treatment), during the stage of rapid fruit growth, CPPU-treated fruit starch concentration had halved (decreased by 1.5 mg gFW^−1^) and glucose concentration increased by about 1.5  mg gFW^−1^. Although from a metabolite point of view the outcome was similar between the two different fruit carbohydrate supplies, transcript analysis suggests that control of carbohydrate metabolism in fruit may have been affected differently than carbohydrate supply to fruit. At a standard carbohydrate supply, transcription of a gene coding for an enzyme critical for starch synthesis (*APL2*) was decreased and transcription of a beta-amylase gene involved in starch degradation (*BAM9*) increased in CPPU-treated fruit. Therefore starch synthesis was reduced and its degradation increased in CPPU-treated fruit, consistent with the overall decrease in starch in these fruit early in fruit development. This is consistent with previous results with kiwifruit, where *BAM9*, coding for a cytosolic enzyme, was associated with starch turnover during early fruit development.^21^ When carbohydrate supply to fruit was high, CPPU-treated fruit showed a general decrease in starch concentration (decreased accumulation of ADP-glucose pyrophosphorylase subunit transcripts) throughout fruit development. These changes in carbohydrate metabolic genes are consistent with observed low starch and high glucose concentration in CPPU-treated fruit.

In CPPU-treated fruit with high carbohydrate supply increased vacuolar invertase *INV3* transcripts suggested an increase in sucrose cleavage, which was also associated with an increase in fructokinase (*FK4*) gene expression in early fruit development. An effect of CPPU stimulating both invertase expression and activity has been observed previously in cucurbitaceae.^9,45,51^ While invertase produces equimolar amounts of glucose and fructose by an irreversible reaction,^52^ the increased concentrations of fructokinase (another irreversible reaction) could be responsible for the phosphorylation of the excess fructose produced, channelling it towards further metabolism. Sucrose synthase, another sucrose cleaving enzyme and fructokinase^53,54^ have high activity early in fruit development.^21^ These two enzymes are both inhibited by fructose so it is important that excess free fructose produced by an increase in invertase cleavage of sucrose is quickly removed via fructokinase. In tomato, transgenic fruit lacking *LeFRK2* transcripts (homologous to kiwifruit *FK4*) had an increased fructose concentration,^55^ and the authors suggested a role for fructokinases in maintaining sugar import into sink tissues.

Later in development (from 70 DAA onwards, after the onset of net starch accumulation), enhancement in glucose and fructose concentrations in CPPU-treated fruit at standard carbohydrate supply was associated by the higher *INV3* transcription. At this time, as a consequence of the general physiological decrease in *FK4* transcription,^21^ fructose also accumulated in fruit and by 126 DAA fructose and glucose had similar concentrations. At high carbohydrate supply, *INV3* transcripts did not differ between CPPU-treated and untreated fruit, although transcripts in fruit from both treatments were elevated by an increase in carbohydrate supply compared with untreated fruit at standard carbohydrate supply. It is possible that the increase in glucose and fructose concentrations in fruit with high carbohydrate supply is determined by the lower *SPSA1* transcription at 70 DAA and *SUS1* down-regulation from 70 DAA onwards, which might have affected sucrose cycling,^56^ and the early onset of fruit maturation suggested by soluble carbohydrate concentrations and earlier *BAM3* transcription.

### CPPU drives fruit expansion by osmotic regulation causing increased water accumulation

The fruit expansion phase, which occurs in kiwifruit from about 50 DAA,^21^ is mainly caused by cell enlargement, due to both increased turgor pressure generated by accumulating osmotically active molecules attracting water and cell wall relaxation.^57^ Vacuolar invertase is a key enzyme for driving cell expansion through osmotic regulation, as shown for cotton fibres’ elongation.^58^ Enhanced glucose (and fructose) in CPPU-treated fruit led to an increase in the soluble carbohydrate component of the fruit osmotic pressure, despite the total osmotic pressure being unchanged in CPPU-treated fruit. This apparent discrepancy could be explained by cellular compartmentalisation: while organic acids are mainly stored in the vacuole,^59^ small soluble carbohydrates can move, actively or passively, between the symplast, apoplast and vacuole, to control cell osmotic potential and turgor pressure.^60^ The movement of soluble carbohydrates between compartments would create an osmotic imbalance likely to attract more water, hence promoting fruit growth through cell expansion. This hypothesis is consistent with the higher water content observed in CPPU-treated fruit.

The higher transcription of transmembrane aquaporin *PIP2.4* at 49 DAA in CPPU-treated fruit is also consistent with the higher water importation inferred in these fruit and driven by the higher glucose and fructose concentrations. Aquaporins control trans-cellular water conductance and have been previously associated with cell expansion.^61^ PIP are divided into two groups: *PIP2*, with high water transport activity ^62^ and *PIP1*, the function of which has been debated as being either non-functional^63^ or requiring a physical interaction and co-expression with *PIP2* to reach the plasma membrane and function as a water channel.^64^ Yanef *et al.*^65^ recently reviewed the complexity of aquaporins and concluded that the PIP1-PIP2 pair should be considered as a functional unit when investigating their role. Our data show that *PIP1.3* transcript accumulation in kiwifruit was delayed and prolonged by CPPU or high carbohydrate availability, and we could speculate that it possibly played a controlling role on the *PIP2.4* water channels, keeping them open for longer and contributing to the higher water uptake of these fruit.

An alternative mechanism for the disproportional increase in water accumulation in CPPU-treated fruit could involve the regulation of *PIP2.4* transcription by sugar signalling, which may discriminate between osmotic pressure generated by soluble carbohydrates or organic acids. This sensing mechanism could be triggered by higher concentrations of glucose, via hexokinase, for example, as hexokinase is a well-known sugar sensor in plants.^66^ A similar interaction between aquaporins and hexokinase has been described in photosynthetic tissues of transgenic tomato plants.^67^ This aspect is worth of further investigation, given the positive and significant correlation between *PIP2.4* and *HK3* transcripts (*P*<0.05; data not shown).

## Conclusion

We have investigated the effects of CPPU on ‘Hayward’ fruit development under controlled standard and high carbohydrate supply to determine whether DM reduction is caused by limited carbohydrate supply to fruit. Despite the presence of high carbohydrate supply, CPPU-treated fruit still showed a dramatic reduction in DM as a consequence of extreme fruit growth osmotically driven by an increase in glucose and fructose concentrations during the early stage of rapid fruit growth. The examination of transcript accumulation of a limited set of candidate genes suggests how changes in carbohydrate metabolism and water uptake in CPPU-treated fruit may occur.

## Figures and Tables

**Table 1:** Effects of CPPU at 7 days after the treatment on fresh weight, dry weight, dry matter (DM) and starch concentration of ‘Hayward’ kiwifruit (35 days after anthesis)

	*Fresh weight (g)*		*Dry weight (g)*		*DM (g kg*^*−1*^)		*Starch concentration (mg gFW*^*−1*^)	
	*Standard*	*High*	*Standard*	*High*	*Standard*	*High*	*Standard*	*High*
Untreated	31.4±1.2	33.2±1.1	2.5±0.1	2.7±0.1	80.7±0.8	81.9±0.8	2.8±0.2	2.5±0.3
CPPU	37.7±1.6	40.4±2.5	2.9±0.1	3.3±0.2	78.2±0.8	80.6±0.8	1.2±0.1	1.3±0.1

*P*-values								
Treatment	**0.001**		**0.005**		**0.028**		**<0.001**	
Carbohydrate supply	0.202		0.105		**0.034**		0.628	
Interaction	0.793		0.648		0.434		0.211	

Abbreviations: ANOVA, analysis of variance; CPPU, cytokinin-like compound forchlorfenuron; DM, dry matter.

Values are averages ±s.e. of the mean. *n*=5 for fresh weight, dry weight and DM; *n*=4 for starch concentration. *P*-values of significant main effects and interactions of fresh weight, dry weight, DM and starch concentration between treatments (untreated or CPPU-treated fruit) and carbohydrate supplies (standard and high) produced using a two-way ANOVA. Significant effects are highlighted in bold.

**Table 2:** DM components during fruit growth in CPPU-treated and untreated fruit at two different carbohydrate supplies (standard and high)

*Treatment*	*Carbohydrate supply*	*Fruit Age (DAA)*	*Starch (% of DM)*	*Soluble carbohydrates (% of DM)*	*Organic acids (% of DM)*	*Others (% of DM)*
Untreated	Standard	35	3.5±0.2	14.8±0.5	22.7±1.3	59.0±1.6
Untreated	Standard	49	5.8±0.3	14.0±0.4	19.2±0.6	61.0±0.9
Untreated	Standard	70	17.9±1.3	7.6±0.4	18.0±0.7	56.5±0.7
Untreated	Standard	126	45.1±3.1	6.8±0.4	14.6±3.4	33.4±3.4
Untreated	Standard	156	46.9±2.1	11.8±0.1	22.4±6.5	18.9±5.2
CPPU	Standard	35	1.5±0.1	17.0±0.6	20.2±0.7	61.3±1.1
CPPU	Standard	49	6.5±0.5	18.1±0.8	17.7±0.8	57.7±1.7
CPPU	Standard	70	18.9±0.8	10.7±0.3	13.5±0.2	56.9±0.7
CPPU	Standard	126	41.9±1.5	10.9±0.1	13.5±0.9	33.7±0.6
CPPU	Standard	156	42.2±1.6	17.3±1.2	12.5±0.9	28.0±1.7
Untreated	High	35	3.0±0.3	14.1±0.2	23.6±2.1	59.2±1.8
Untreated	High	49	4.8±0.4	16.5±1.0	18.6±0.4	60.1±0.9
Untreated	High	70	18.7±1.7	7.4±0.6	15.5±0.6	58.4±1.3
Untreated	High	126	40.7±3.5	6.6±0.6	10.3±1.7	42.5±4.9
Untreated	High	156	40.8±1.1	13.7±0.5	12.7±1.7	32.8±3.0
CPPU	High	35	1.6±0.1	15.9±0.7	19.0±1.4	63.5±1.9
CPPU	High	49	5.5±0.8	19.9±0.4	17.0±0.7	57.6±1.4
CPPU	High	70	20.9±1.6	12.7±0.6	13.7±0.6	52.8±1.7
CPPU	High	126	46.0±3.6	10.5±0.5	13.0±1.1	30.5±3.4
CPPU	High	156	38.2±3.1	17.3±1.0	15.8±4.1	28.6±0.8
						
P*-values*						
Treatment			0.619	**<0.001**	**0.026**	0.291
Carbohydrate supply			0.203	**0.043**	0.116	0.067
Treatment×carbohydrate supply			0.121	0.707	0.074	**0.008**
Fruit age			**<0.001**	**<0.001**	**<0.001**	**<0.001**
Fruit age×treatment			0.202	**0.046**	0.562	**0.038**
Fruit age×carbohydrate supply			0.135	**0.009**	0.838	0.101
Fruit age×treatment×carbohydrate supply			0.447	0.241	0.123	0.071

Abbreviations: ANOVA, analysis of variance; CPPU, cytokinin-like compound forchlorfenuron; DAA, days after anthesis; DM, dry matter.

DM components (starch, soluble carbohydrates, organic acids and other non-detected compounds) are reported as percentage of total DM. Values are averages ±s.e. of the mean. *n*=4. DAA, days after anthesis. *P*-values of significant main effects and interactions of starch, soluble carbohydrates, organic acids and other non-detected compounds between treatments (untreated or CPPU treated fruit), carbohydrate supplies (standard and low) and fruit age produced using a three-way factorial ANOVA. Significant effects are highlighted in bold.

**Table 3:** *P*-values of main effects and interactions of total fruit osmotic pressure and its components between treatments (untreated or CPPU treated fruit), carbohydrate supplies (standard and high) and fruit age (days after anthesis) produced using a three-way factorial ANOVA

P*-values*	*Total fruit osmotic pressure*	*Soluble carbohydrates fruit osmotic pressure*	*Organic acids fruit osmotic pressure*
Treatment	0.378	**<0.001**	**0.011**
Carbohydrate supply	0.757	**0.001**	0.299
Treatment×carbohydrate supply	0.064	0.686	**0.050**
Fruit age	**<0.001**	**<0.001**	**<0.001**
Fruit age×treatment	0.641	0.077	0.517
Fruit age×carbohydrate supply	0.902	**0.003**	0.871
Fruit age×treatment×carbohydrate supply	0.164	0.237	0.091

Abbreviations: ANOVA, analysis of variance; CPPU, cytokinin-like compound forchlorfenuron.

Effects with *P*<0.05 are highlighted in bold.
